# Sex, early life adversity, and negative self-evaluation shape the association between negative life events and depressive symptoms in adolescence

**DOI:** 10.1038/s41598-025-29337-z

**Published:** 2025-12-05

**Authors:** Kate Ryan Kuhlman, Elizabeth E. Antici, Haley Dveirin, Mai-Lan M. Tran, Natalie A. Hall, Paul Delacruz, Julienne E. Bower

**Affiliations:** 1 Department of Psychology, School of Social Ecology, University of California Irvine, Irvine, CA 92697 USA; 2 Institute for Interdisciplinary Salivary Bioscience, University of California Irvine, Irvine, CA 92697 USA; 3 Cousins Center for Psychoneuroimmunology, Semel Institute for Neuroscience and Human Behavior, University of California Los Angeles, Los Angeles, CA 90097 USA; 4 Department of Psychology, University of California Los Angeles, Los Angeles, CA 90097 USA

**Keywords:** Stress, Depression, Adolescence, Early life adversity, Sex differences, Psychology, Human behaviour

## Abstract

Adolescent depression is an increasing public health concern. Recent experiences of negative events are associated with an increase in depressive symptoms and onset of major depression, but how factors such as sex and early life adversity (ELA) influence this association remains unclear. Data included 388 observations comprised of self-reported negative events and depressive symptoms measured every 4 months across a 12-month period by 97 adolescents oversampled for ELA and aged 11–17 (46.4% female). Higher between-person averages in negative events were associated with greater total depressive symptoms, specifically dysphoric mood and somatic complaints. Within-person variability in negative events was not associated with total depressive symptoms or any symptom subscales. Females with higher between-person negative events reported larger increases in negative self-evaluation symptoms than males. Among adolescents with high ELA exposure, higher between-person negative events were associated with more total depressive symptoms and increasing symptoms over time. The present data supported the well-established association between negative events and depressive symptoms, particularly negative self-evaluation symptoms and among females. Data support efforts to prevent depression among ELA-exposed adolescents regardless of ongoing stress exposure as well as sex-specific symptom targets that may mitigate risk.

## Introduction

Depression is a leading contributor to the global burden of disease, with a large personal, societal, and economic cost^1^. There is a large literature showing that stress contributes to depression^2–4^, with more recent reviews asserting a continued need to understand risk factors for first-onset and models that account for the differential susceptibility to depression in the context of stress among females. Risk for psychiatric disorders, including major depressive disorder (MDD), rises rapidly during adolescence^5,6^. Indeed, the prevalence of depression among 12-year-olds is 4% but rises to almost 14% by age 15^7^. The present study aimed to advance our understanding of how stress contributes to depression-onset by modeling the association between negative life events and depressive symptoms as measured serially across a 12-month period in a sample of adolescents oversampled for early life adversity (ELA; a risk factor for depression onset in adolescence) but without any history of MDD.

Negative life events are consistently associated with an increase in depressive symptoms, and are robust predictors of major depression onset^2–4,8^. Negative life events include undesirable, severe incidents such as serious threats to core relationships or occupation, or significant negative changes to finances or health^9^. Those who develop depression are 2.5 to 9.4 times more likely to have faced a major negative life event before their first episode of depression^4,10^. Not all those who experience negative life events develop depression, though adolescents may be more vulnerable to this pathway to depression. The simultaneous development of neural, biological, and psychosocial functions makes adolescence a period of increased sensitivity to both negative and positive experiences^11^. Further, many negative life events are either social in nature (e.g., end of a relationship) or have social implications (e.g., moving homes or schools), and adolescents are more sensitive to social stimuli, social evaluation, social deprivation, and peer rejection^12–15^. Understanding which adolescents are at the greatest risk of experiencing depression and its symptoms in the aftermath of stressful life events will guide prevention and early intervention efforts that mitigate the links between stress and depression with precision.

One hypothesis surrounding which adolescents are most at risk for developing depression following stressors is guided by the stress sensitization hypothesis. The stress sensitization hypothesis posits that cumulative exposure to negative life events during childhood predisposes individuals to the development of psychopathology following later stressful life events^2,3,16^. There is robust empirical evidence for the stress sensitization hypothesis, showing that ELA increases risk for depression, PTSD, and anxiety disorders following stressful life events in adulthood^17–19^. ELA also predicts greater depression and externalizing problems among adolescents exposed to recent stress^20–22^. Although this literature supports that individuals with a history of ELA are at heightened risk for developing psychopathology in the wake of later stress, it focuses on between-person associations, which compare individuals with more recent stress to individuals with less. These approaches leave unknown whether the effects of stress on depressive symptoms occur as a function of between-person differences in stress exposure or within-person differences in sensitivity to those exposures.

To our knowledge, only three studies to date have examined whether stress sensitization is reflected within individuals over time^23–25^. In one study, adolescents who reported greater average stressful life events over a 2-year study period also endorsed more severe depressive symptoms (between-person effect)^23^. These adolescents also reported more elevated depressive symptoms at times when they experienced more recent stress than at other time points^23^. Another longitudinal study found that young adults with greater childhood emotional abuse report greater within-person increases in depressive symptoms in the aftermath of recent stress^25^. Lastly, a third longitudinal study aiming to address the preponderance of depression among adolescent females showed that females evidence greater within-person sensitization to depressive symptoms than males following increases in bullying^24^. These findings generally support the stress sensitization hypothesis and the role of within-person stress sensitization, and introduce the hypotheses that within-person sensitization may be specific to adolescents who are female or those with a history of ELA. The present study aims to test these hypotheses directly.

Further, the vast majority of research on links between stress and depression onset preceded dimensional approaches to psychopathology (e.g., RDoC) which seek to identify transdiagnostic, biobehavioral characteristics that precede the emergence of a behavioral disorder [e.g., 26,27]. While depression is a heterogenous disorder, clusters of symptoms within depression have been identified as promising endophenotypes and modifiable treatment targets, such as anhedonia and attributional biases. Previous studies have not investigated whether sensitization to stressful life events varies across symptom domains. Given the well-established links between stress and multiple forms of psychopathology and that depressive symptoms occur in many psychiatric disorders^28^, characterizing links between stress and symptom domains is essential to transdiagnostic generalizability.

The present study aimed to extend the current literature on stress and depression by examining both between-person and within-person variability in negative events and depressive symptoms assessed serially over a 12-month period among depression-naïve adolescents oversampled for ELA. We hypothesized that exposure to more negative events over the study period would be associated with more depressive symptoms (between-person), that adolescents would report the highest depressive symptoms at assessments when they also reported experiencing more recent negative events (within-person), and that these associations would be stronger among female adolescents and those exposed to more ELA.

## Method

### Participants

Participants in this sample were 97 adolescents enrolled in the Teen Resilience Project, a prospective and longitudinal study of the biobehavioral factors linking ELA to depression-onset in adolescents. Adolescents were 46.39% female with an average age of 13.91 years (*SD* = 1.59); 72.16% of participants identified as Non-Hispanic White, 26.80% as Hispanic, 17.53% as Asian, 10.31% as Black/African American, 3.09% as Native Hawaiian or other Pacific Islander, and 2.06% as American Indian/Alaskan Native (groups were not mutually exclusive). Participants were recruited via mass mailing based on census records targeting households with adolescents. These mass mailings included a letter inviting parents to contact our team to learn more. Parents who contacted the study team completed a phone interview to determine eligibility. Youth were eligible to participate if they were between the ages of 11–17. Because the parent study aimed to understand immune and endocrine mechanisms in depression-onset among ELA-exposed youth, youth were not eligible to participate if they could not read or understand English; had a bleeding disorder (e.g., hemophilia); had any current or past major depressive episodes, psychotic symptoms, mania, autism spectrum disorder, or any current chronic medical conditions (e.g., cancer, diabetes); or were regularly taking medications known to influence the immune system (e.g., antihistamines, inhaler, antidepressants).

### Procedures

All study procedures were approved by the Institutional Review Boards at the University of California, Los Angeles and University of California, Irvine and were conducted in accordance with these approved procedures. Participants were recruited to participate in a prospective, longitudinal study on the biobehavioral predictors of depression among adversity-exposed adolescents^29,30^. Interested parents completed a semi-structured screening interview over the phone to determine eligibility. Eligible participants and their parents/guardians came into the laboratory to provide written and informed consent and assent before enrolling in the study. Study enrollment visits occurred between June 2017 and February 2020. At these visits, participants completed questionnaires on their demographics, as well as self-reported measures of negative events and depressive symptoms. Four, eight, and 12 months after their enrollment visit, participants received a text and email that included a link to complete a battery of follow-up questionnaires, including those on negative events and depressive symptoms. Participants received $60 at the end of the study enrollment visit, as well as $15 for each follow-up assessment they completed. All 97 participants enrolled in the study completed all follow-up assessments (100% retention). At the end of the 12-month period, participants and their parents completed a semi-structured diagnostic interview^31^ over the phone to assess the occurrence of major depressive episodes over the past year. All interviews were conducted by a licensed clinical psychologist or an advanced doctoral student supervised by a licensed clinical psychologist. Institutional restrictions on research activities caused recruitment and enrollment in this study to stop in March 2020 as a result of the COVID-19 pandemic, however electronic follow-up assessments continued uninterrupted. Of the 388 total observations, 93 (23.7%) occurred after the beginning of the COVID-19 pandemic (March 11, 2020), and study enrollment was closed indefinitely as a result of state and institutional precautions taken to slow the spread of the virus.

### Measures

#### Negative events

Negative events were assessed every four months for 12 months using an 18-item checklist developed from previous studies linking stressful life events across multiple domains (family, friends, school) to negative health outcomes^32–34^. The items and their frequencies of endorsement in this sample are summarized in Table 1. For each item, participants indicated whether the event occurred in the past 4 months by answering “yes” or “no”. The total number of “yes” responses were summed to produce their score. Every item was endorsed at least once across the time frame. The most frequently endorsed items (items endorsed by 15% or more participants during at least 1 time point) included: did not see your father for 1 month or longer; serious falling out with a friend; physical appearance got worse; and close family member, friend, or pet passed away.

**Table 1:** Negative events.

Item	Frequency *n* (%)
Did not see your mother for one month or longer.	15 (15.5)
Did not see your father for one month or longer.	24 (24.7)
You became seriously ill.	9 (9.3)
A sister or brother had a drug or alcohol problem.	6 (6.2)
A close family member or friend was arrested.	0 (0.0)
A close friend moved quite far away.	18 (18.6)
You had a serious falling-out or ended a friendship with a close friend.	35 (36.1)
Your grades in school went down a lot.	25 (25.8)
You were suspended or expelled from school.	2 (2.1)
You did not get into a club, sports team, or school you really wanted to be in.	14 (14.4)
Your physical appearance got worse (e.g., weight, acne, etc.)	40 (41.2)
A parent lost his/her job.	7 (7.2)
Your parents divorced or separated	6 (6.2)
A close family member, friend, or important pet passed away.	24 (24.7)
Your family experienced serious financial problems.	9 (9.3)
An immediate family member became seriously ill.	19 (19.6)
Your family moved to a new house.	2 (2.1)
You changed schools.	12 (12.4)

#### Depressive symptoms

Depressive symptoms were measured via the Reynolds Adolescent Depression Scale - Second Edition (RADS-2)^35,36^ at each assessment (every four months throughout the 12-month period). The RADS-2 consisted of a 30-item checklist asking participants to rate how they had been feeling over the past month on a 4-point Likert-type scale, with 1 being “almost never” and 4 being “most of the time.” The RADS-2 is composed of four subscales: dysphoric mood, anhedonia, somatic complaints, and negative self-evaluation. The dysphoric mood subscale consisted of 8 items such as “I feel lonely” and “I feel like crying”. The anhedonia subscale consisted of 7 reverse-scored items such as “I feel like having fun” and “I feel like talking to other students”. The somatic complaints subscale consisted of 7 items such as “I feel sick” and “I feel tired.” The negative self-evaluation subscale consisted of 8 items such as “I feel my parents don’t like me” and “I feel that no one cares about me.” A total score with all 30-items was computed, as well as individual scores for each of the four subscales. Higher scores indicated more severe depressive symptoms. The RADS-2 demonstrated excellent reliability in this sample at all four timepoints, *α* = 0.93–0.94. The dysphoric mood subscale demonstrated good reliability in this sample at all four timepoints, *α* = 0.87–0.89. The anhedonia subscale demonstrated acceptable reliability in this sample at all four timepoints, *α* = 0.73–0.82. The somatic complaints subscale demonstrated acceptable reliability in this sample at all four timepoints, *α* = 0.78–0.83. The negative self-evaluation subscale demonstrated good reliability in this sample at all four timepoints, *α* = 0.88–0.90. Total scores on the RADS-2 ≥ 77 indicate clinically elevated depressive symptoms, which showed moderate convergent validity with clinician-administered diagnostic interviews at the 12-month follow-up assessment.

#### Moderators: sex and ELA

Participants indicated their sex at the study enrollment visit via a self-report questionnaire. Participants responded to the question “what is your sex?” and selected either “male” or “female”. ELA was measured at study enrollment via a composite of the child-reported Early Trauma Inventory (ETI)^37,38^, child-reported Risky Families Questionnaire (RFQ)^39^, and a parent-reported adverse childhood experiences (ACE) questionnaire^40^. The use of a composite is consistent with best practices in assessment of stress^41^ as well as child and adolescent psychopathology^42^ because these three measures vary with respect to the reporter and the response options, allowing balance across the limitations of each. The ETI included 37 items separated into three subscales: physical abuse, emotional abuse, and general abuse, as well as one additional question about sexual abuse/assault. Each item asked whether a potentially traumatic event had ever happened to the participant, to which they could respond with “yes” or “no.” “Yes” responses were summed. The RFQ included 13 items rated on a 5-point Likert scale with a 0 representing “not at all” to a 4 representing “very often.” Example items included, “How often did a parent or other adult in the household swear at you, insult you, put you down, or act in a way that made you feel threatened?” and, “How often would you say you were neglected while you were growing up, that is, left on your own to fend for yourself?” RFQ internal reliability was good in this sample, *α* = 0.80. The ACE questionnaire included 9 items in which parents were asked to answer “yes” or “no” to whether potential adverse events had occurred during their child’s lifetime such as parental separation or divorce, death of a close relative, and living in poverty. Total ETI, RFQ, and ACE scores were standardized into *z*-scores and then averaged to create a composite ELA index where higher values indicated more ELA, and values greater than 1 reflected participants for whom exposure to ELA was at least one standard deviation greater than the average of participants in the sample.

#### Data analysis

All data analyses were conducted within SPSS v. 28 using linear mixed models. All mixed models used an unstructured covariance matrix and included a random intercept and slope of time. All models also used maximum likelihood estimation. Interpretation of model results emphasized estimate magnitude, precision, and sensitivity. With 4 observations per participant (*n* = 97), assuming a two-tailed *α* = 0.05 and power = 0.80, the study design could detect standardized effects of small-to-moderate size (approximately 0.28 SD) for between-person predictors and small (approximately 0.16 SD) for within-person predictors. Rather than report dichotomous significance, we report coefficient estimates and their 95% confidence intervals to aid interpretation of reliability. Statistical significance was inferred if bootstrapped confidence intervals did not contain zero, however *p*-values are also reported and a *p*-value of 0.01 or below survives correction for multiple comparisons across the 5 outcomes.

The primary outcome was total depressive symptoms, and secondary outcomes were depressive symptom subscales: dysphoric mood, anhedonia, somatic complaints, and negative self-evaluation. A preliminary model was used to determine whether a linear or quadratic model best fit the trajectory of total depressive symptoms across the 12 months. A linear model was a better fit to the depressive symptom data than a quadratic model and was used for all subsequent models. Models were first conducted unadjusted, then adjusted for a priori covariates: age (in years), sex, and whether the assessment occurred during the COVID-19 pandemic (after March 11, 2020). Results of unadjusted models are reported in text and results of adjusted models are reported in Table 3. Psuedo-R^2^ indices were computed by comparing model fit (AIC) with and without fixed effects. Main effect models predicted symptom outcomes as a function of the fixed effects of time (in months), between-person average negative events and within-person variability in negative events, and their interactions. Moderations by sex and ELA were then tested by predicting symptom outcomes as a function of the fixed effects of time (in months), between- and within-person variability in negative events, sex or ELA, their 2-way interactions, and their 3-way interactions.

## Results

Table 2 provides summary characteristics about the participants, their exposure to negative events, and their depressive symptoms. Negative events were common in this sample; participants reported experiencing at least one negative event at 71.1% of observations and between 0 and 8 negative events across the study period. On average, participants reported moderate to high depressive symptoms over the 12-month follow-up period. At each assessment timepoint, between 20.2% and 29.8% of participants reported clinically elevated depressive symptoms and 13.4% (*n* = 13) of the sample were identified as experiencing a depressive episode at some point during the 12 months based on diagnostic interview.

**Table 2:** Participant characteristics and descriptive statistics (*n* = 97).

		Mean (SD)	*n* (%)
Age		13.91 (1.59)	
Sex (% female)			45 (46.39)
Race/ethnicity^1^	American Indian / Alaskan Native		2 (2.06)
	Asian		17 (17.53)
	Black / African American		10 (10.31)
	Hispanic		26 (26.80)
	Native Hawaiian or Other Pacific Islander		3 (3.09)
	White		70 (72.16)
Negative events	Baseline	1.42 (1.26)	
	4-month follow-up	1.53 (1.66)	
	8-month follow-up	1.27 (1.54)	
	12-month follow-up	1.37 (1.44)	
Depressive symptoms^2^	Baseline	59.38 (17.79)	19 (19.59) ^**2**^
	4-month follow-up	63.29 (18.10)	24 (24.74) ^**2**^
	8-month follow-up	61.91 (18.19)	23 (23.71) ^**2**^
	12-month follow-up	64.44 (18.65)	28 (28.87) ^**2**^
Early life adversity	ACE	2.11 (1.57)	
	Risky Families Questionnaire	11.18 (7.42)	
	Early Trauma Inventory	9.59 (5.43)	
Household income	Less than $50,000 per year		13 (13.40)
	$50,000 to $100,000 per year		19 (19.60)
	More than $100,000 per year		63 (64.90)

**Table 3:** Estimated depressive symptoms and subscales over 12-month follow-up by negative events after adjusting for key covariates.

	Total		Dysphoric mood		Anhedonia		Somatic complaints		Negative Self-evaluation	
AIC	2,825.30		2,003.62		2,188.80		1,860.20		1,989.41	
	b (SE)	95%CI	b (SE)	95%CI	b (SE)	95%CI	b (SE)	95%CI	b (SE)	95%CI
Intercept	60.04 (2.40)***	55.29, 64.79	17.14 (0.73)***	15.70, 18.58	14.68 (0.88)***	12.94, 16.43	14.96 (0.59)***	13.79, 16.12	13.27 (0.68)	11.92, 14.62
Time	0.50 (0.20)*	0.06, 0.92	0.19 (0.06)**	0.06, 0.32	0.07 (0.06)	-0.12, 0.25	0.19 (0.05)***	0.08, 0.29	0.04 (0.06)	-0.08, 0.17
Person-level NEs	3.11 (1.34)*	0.46, 5.77	0.76 (0.41)+	-0.05, 1.57	0.54 (0.49)	-0.44, 1.52	0.64 (0.33)+	-0.01, 1.29	1.17 (0.38)**	0.42, 1.93
Within-person NEs	-2.42 (1.71)	-5.79, 0.95	0.004 (0.57)	-1.20,1.10	-1.33 (0.74)	-2.79, 0.13	-0.77 (0.47)	-1.69, 0.15	− 0.74 (0.57)	-1.86, 0.37
Person-level NEs*time	0.04 (0.12)	-0.20, 0.27	-0.01 (0.03)	-0.07, 0.06	0.04 (0.05)	-0.06, 0.14	-0.01 (0.03)	-0.06, 0.05	0.011 (0.34)	-0.06, 0.08
Within-person NEs*time	0.26 (0.21)	-0.16, 0.67	-0.01 (0.07)	-0.15,0.12	0.15 (0.09)	-0.03, 0.32	0.08 (0.06)	-0.04, 0.18	0.10 (0.07)	-0.04, 0.24
Age	3.27 (1.04)**	1.20, 5.34	1.19 (0.32)***	0.56, 1.82	0.73 (0.38)	-0.04, 1.48	0.64 (0.26)*	0.13, 1.15	0.70 (0.30)*	0.11, 1.29
Sex	-0.73 (3.29)	-7.23, 5.79	-8.21 (1.00)	-2.80, 1.16	-0.98 (1.21)	-2.49, 2.30	0.35 (0.81)	-1.25, 1.95	-0.11 (0.94)	-1.97, 1.75
COVID-19	5.62 (2.99)+	-0.27, 11.51	2.63 (1.02)*	0.63, 4.63	1.02 (1.31)	-1.56, 3.60	1.01 (0.83)	-0.54, 2.74	0.44 (1.02)	-1.56, 2.45
Age*time	0.23 (0.90)	-0.16, 0.20	-0.01 (0.03)	-0.06, 0.040	-0.003 (0.04)	-0.08, 0.07	0.02 (0.02)	-0.02, 0.06	0.02 (0.03)	-0.03, 0.07
Sex*time	-0.24 (0.28)	-0.81, 0.32	-0.13 (0.08)	-0.29, 0.03	0.03 (0.12)	-0.22, 0.27	-0.13 (0.07)*	-0.26, − 0.002	-0.02 (0.08)	-0.18, 0.14
COVID-19*time	-0.33 (0.32)	-0.97, 0.31	0.14 (0.11)	-0.35, 0.08	-0.05 (0.14)	-0.33, 0.23	-0.09 (0.09)	-0.26, 0.08	0.001 (0.11)	-0.21, 0.21

Note: ***p < .001, **p < .01, *p < .05, +*p* < .10

### Change in depressive symptoms over 12 months

Unadjusted models showed that depressive symptoms increased across the 12-month study period, *b* = 0.41 (*SE* = 0.14), *95%CI*: 0.13–0.69, *p* = .004. This increase over time was specific to dysphoric mood, *b* = 0.16 (*SE* = 0.04), *95%CI*: 0.08–0.24, *p* < .001, and somatic complaints, *b* = 0.12 (*SE* = 0.03), *95%CI*: 0.05–0.18, *p* = .001, with no increase over time observed for anhedonia, *p* = .13, or negative self-evaluation, *p* = .28. There remained a significant increase in depressive symptoms across the follow-up period after accounting for age, sex, and whether the assessment took place after the start of the COVID-19 pandemic, *b* = 0.48 (*SE* = 0.22), *95%CI*: 0.04–0.91, *p* = .03. There also remained a significant increase in dysphoric mood and somatic complaints after accounting for age and sex, and whether the assessment occurred during the first year of the COVID-19 pandemic.

### Unadjusted main effects of negative events on depressive symptoms

Negative events accounted for 4.20% of additional variance in total depressive symptoms (AIC without negative events in the model = 2,978.74 vs. AIC with negative events in the model = 2,853.47). Depressive symptoms in this sample varied largely as a function of between-person differences in negative events. Specifically, between-person average negative events were associated with higher total depressive symptoms, *b* = 4.05 (*SE* = 1.34), *95%CI*: 1.39–6.71, *p* = .003, but not with increases in total depressive symptoms over time, *b* = 0.07 (*SE* = 0.11), *95%CI*: -0.16 to 0.29, *p* = .56. Within-person variability in negative events was not associated with total depressive symptoms, *b* = -2.27 (*SE* = 1.71), *95%CI*: -5.63 to 1.10, *p* = .19, or their change over time, *b* = 0.23 (*SE* = 0.21), *95%CI*: -0.19 to 0.64, *p* = .28.

Negative events accounted for between 4.15 and 5.00% of variance in depressive symptom subdomains. Negative events accounted for 4.30% of additional variance in dysphoric mood (AIC_without NE_ = 2125.51 vs. AIC_with NE_ = 2,034.09), 5.00% of additional variance in anhedonia (AIC_without NE_ = 2315.12 vs. AIC_with NE_ = 2200.29), 4.15% of additional variance in somatic complaints (AIC_without NE_= 1966.41 vs. AIC_with NE_ = 1884.87), and 4.17% of additional variance in negative self-evaluation (AIC_without NE_ = 2090.18 vs. AIC_with NE_ = 2003.08).

Before accounting for age, sex, and whether the assessment occurred during COVID-19, between-person average negative events were associated with higher dysphoric mood, *b* = 1.13 (*SE* = 0.41), *95%CI*: 0.31–1.96, *p* = .007, somatic complaints, *b* = 0.81 (*SE* = 0.32), *95%CI*: 0.17–1.45, *p* = .01, and negative self-evaluation, *b* = 1.37 (*SE* = 0.38), *95%CI*: 0.62–2.12, *p* < .001. Between-person average negative events were not associated with anhedonia, *b =* 0.73 (*SE* = 0.49), *95%CI*: -0.23-1.70], *p* = .13. Between-person average negative events were not associated with changes over time in dysphoric mood, *b* = 0.003 (*SE* = 0.03), *95%CI*: -0.06 to 0.07], *p* = .93, anhedonia, *b =* 0.04 (*SE* = 0.05), *95%CI*: -0.06 to 0.14, *p* = .43, somatic complaints, *b* = 0.005 (*SE* = 0.03), *95%CI*: -0.49 to 0.06, *p* = .85, or negative self-evaluation, *b* = 0.02 (*SE* = 0.03), *95%CI*: -0.05 to 0.08], *p* = .56. Within-person variability in negative events was not associated with any symptom subscales [dysphoric mood: *b* = 0.13 (*SE* = 0.58), *95%CI*: -1.01 to 1.26, *p* = .83; anhedonia: *b* = -1.13 (*SE* = 0.73), *95%CI*: -2.57 to 0.31], *p* = .12; somatic complaints: *b* = -0.87 (*SE* = 0.47), *95%CI*: -1.8 to 0.06, *p* = .07; negative self-evaluation: *b* = -0.74 (*SE* = 0.56), *95%CI*: -1.85 to 0.36, *p* = .19] or their change over time [dysphoric mood: *b* = -0.03 (*SE* = 0.07), *95%CI*: -0.17 to 0.11], *p* = .67; anhedonia: *b* = 0.12 (*SE* = 0.09), *95%CI*: -0.05 to 0.30], *p* = .17; somatic complaints: *b* = 0.08 (*SE* = 0.06), *95%CI*: -0.03 to 0.20], *p* = .15; negative self-evaluation: *b* = 0.10 (*SE* = 0.07), *95%CI*: -0.04 to 0.23, *p* = .16].

### Adjusted main effects of negative events on depressive symptoms

Estimated associations between negative events and depressive symptoms when accounting for covariates are shown in Table 3. Most notably, after accounting for age, sex, and COVID-19, between-person average negative events were only associated with higher negative self-evaluation.

### Sex moderated the effect of negative events on depressive symptoms

Sex was not associated with total depressive symptoms, dysphoric mood, anhedonia, or any symptom domains, all *p* > .39, nor their change over time, all *p* > .10. While sex was not associated with differences in somatic complaints at study enrollment, *p* = .63, males did report decreasing somatic complaints across the study period, *b* = -0.15 (*SE* = 0.07), *95%CI*: -0.28 to -0.14], *p* = .03. In addition to these main effects of sex on depressive symptoms, sex moderated the between-person association of negative events with negative self-evaluation, *b* = -1.78 (*SE* = 0.73), *95%CI*: -3.23 to -0.33, *p* = .02, but did not moderate change in negative self-evaluation over time, *b* = 0.03 (*SE* = 0.07), *95%CI*: -0.10 to 0.17, *p* = .62. Sex did not moderate the within-person association between negative events and negative self-evaluation, or their change over time, all *p*s > 0.16.

**Fig. 1:**
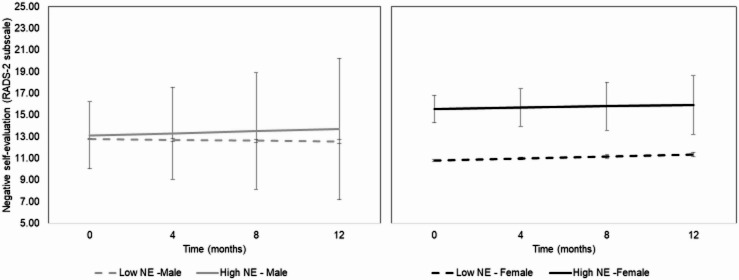
Estimated symptoms of negative self-evaluation by between-person negative events and sex.

Because there was a significant interaction at the between-person level of analysis, models were then fit separately for male and female participants to estimate simple slopes for each group. These models support greater susceptibility to negative self-evaluation symptoms in the context of negative events. Specifically, among females, higher between-person negative events were associated with more negative self-evaluative symptoms, *b* = 2.01 (*SE* = 0.52), *95%CI*: 0.97–3.06, *p* < .001, but not their change over time, *b* = -0.01 (*SE* = 0.05), *95%CI*: -0.10 to 0.09, *p* = .90. Further, among females, within-person variability in negative events was inversely associated with negative self-evaluation, *b* = -1.80 (*SE* = 0.85), *95%CI*: -3.49 to -0.11, *p* = .04, but not their increase over time, *b* = 0.19 (*SE* = 0.10), *95%CI*: -0.02 to 0.39, *p* = .07. Between and within-person variability in negative events were not associated with negative self-evaluation or its change over time among males, all *p*s > 0.30. See Fig. 1 for estimated negative self-evaluation symptoms by between-person negative events and sex.

Sex did not moderate the between- or within-person associations between negative events and depressive symptoms (total or dysphoric mood, anhedonia, or somatic complaints subscales), or their change over the 12-month follow-up period, all *p*s > 0.23.

### ELA moderated the association between between-person negative events and depressive symptoms

**Fig. 2:**
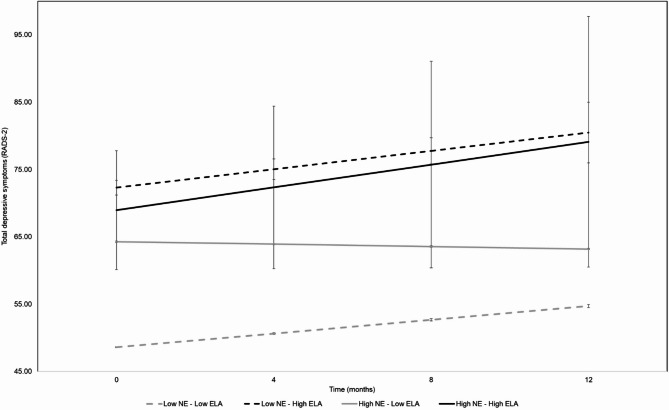
Estimated total depressive symptoms by between-person variability in negative events and ELA.

ELA was associated with more total depressive symptoms, *b* = 7.18 (*SE* = 2.45) *95%CI*: 2.31–12.04, *p* = .004, but not their change over time, *b* = 0.28 (*SE* = 0.22), *95%CI*: -0.17 to 0.72, *p* = .22. ELA moderated the association between person-level negative events and total depressive symptoms, *b* = -3.80 (*SE* = 1.58), *95%CI*: -6.94 to -0.67, *p* = .02, but not change in depressive symptoms over time, *b* = 0.15 (*SE* = 0.14), *95%CI*: -0.13 to 0.44, *p* = .29. See Fig. 2 for estimated total depressive symptoms over the 12-month follow-up by between-person negative events and ELA. Specifically, among participants with low exposure to ELA, higher between-person negative events were associated with more total depressive symptoms, *b* = 6.52 (*SE* = 2.66), *95%CI*: 1.18–11.86, *p* = .02, and an attenuated increase in symptoms over time, *b* = -0.39 (*SE* = 0.17), *95%CI*: -0.74 to -0.04, *p* = .03. ELA did not moderate the association of within-person negative events with depressive symptoms, nor with change over time in depressive symptoms, *p*s > 0.29.

ELA was also associated with significantly higher somatic complaints, *b* = 1.73 (*SE* = 0.60), *95%CI*: 0.55–2.91], *p* = .005, and more negative self-evaluation, *b* = 2.68 (*SE* = 0.69), *95%CI*: 1.30–4.06], *p* < .001, but not dysphoric mood, *b* = 1.48 (*SE* = 0.77), *95%CI*: -0.04 to -3.00, *p* = .06, nor anhedonia, *b* = 1.32 (*SE* = 0.94), *95%CI*: -0.54 to 3.18, *p* = .16. ELA moderated the between-person negative events and dysphoric mood association, *b* = -1.06 (*SE* = 0.49), *95%CI*: -2.03 to -0.08, *p* = .04, as well as associations with anhedonia *b* = -1.24 (*SE* = 0.60), *95%CI*: -2.44 to -0.04, *p* = .04, and somatic complaints *b* = -1.07 (*SE* = 0.38), *95%CI*: -1.83 to -0.31, *p* = .006, but not negative self-evaluation, *b* = -0.44 (*SE* = 0.45), *95%CI*: -1.32 to 0.45, *p* = .33.

## Discussion

The present study aimed to extend the existing literature on stress and depression by examining the role of both between- and within-person variability in negative events and depressive symptoms among adolescents over-sampled for ELA across a 12-month longitudinal follow-up period. Depressive symptoms rose across the 12 months, specifically dysphoric mood and somatic complaints whereas no increases in anhedonia or negative self-evaluation were observed. More between-person negative events were associated with higher total depressive symptoms as well as negative self-evaluation. Contrary to our hypotheses, there was little to no evidence of within-person sensitivity for total depressive symptoms or any of the symptom domains. Sex moderated the stress-depressive symptom link such that more negative events were associated with more negative self-evaluation symptoms among females but not males. We also found that ELA moderated the between-person negative events and total depressive symptoms association. Specifically, adolescents with low ELA reported higher total depressive symptoms in the context of higher than average negative events. Together, these findings guide efforts to better understand how longitudinal links between negative events and depressive symptoms can be translated into targeted interventions.

Data from the present sample supported a strong between-person association between life events and depression, such that adolescents exposed to the highest average negative events over 12 months also reported the most depressive symptoms. While prior research has demonstrated a robust effect of between-person negative events on the development of depression^2,3^, the study by Jenness and colleagues (2019) was one of few to characterize this effect at both the within- and between-person levels of analysis. They found that at times when adolescents experienced more negative events than was typical for them, they reported more depressive symptoms than usual, as well as replicated previous findings for between-person negative events. Additionally, their results supported the stress sensitization hypothesis, showing that the within-person association between negative events and depression was even stronger among adolescents who experienced higher average negative events across the 2-year study period. The between-person associations observed in the present data mirror these between-person effects, but diverge from the within-person effects. Several differences between the current study and that of Jenness and colleagues (2019) may have contributed to these differential findings. First, the Jenness and colleagues (2019) measure of negative events included 57 items at each assessment, rated on a Likert-type scale to reflect how often an event occurred from *never* to *almost always*. Adolescents in the present sample simply affirmed or denied their exposure to a checklist of 18 events. The adolescents reported between 1 and 2 negative events during each 4-month assessment interval, which was comparable to other adolescent samples assessed using this same measure^43^. Differences in study design, including length of study period, number of data collection points, sample size, and assessment of negative events could also have contributed to differences in results. Lastly, demographic differences could have contributed to the differential findings. For example, the current study intentionally oversampled adolescents with ELA^29^, and recruited participants from the Southern California region, which included a higher percentage of Hispanic and Asian participants, whereas the prior study recruited adolescents from Chicago, Illinois, and Montreal, Quebec, Canada and included a higher proportion of Black participants. A large, nationally representative study of negative events and depressive symptoms may be warranted to determine whether within-person effects of negative events on depressive symptoms are driven by frequency or chronicity of negative events as opposed to their mere occurrence, or vary by race, ethnicity, or other demographic factors.

Sex moderated the between-person association between negative events and the negative self-evaluation subscale of depressive symptoms assessed across the 12-month study period. More reports of negative events were associated with greater negative self-evaluation scores among females, but not males. This was consistent with existing literature suggesting that females are more susceptible to depression during adolescence^44,45^, prone to depressive symptoms in the aftermath of bullying^24^, and vulnerable to internalizing negative experiences^46,47^. Previous studies have highlighted gender differences in emotional processing and coping strategies, indicating that females often report more rumination and self-focused negative thoughts than males^48^. The present results extended these findings by highlighting specificity to symptoms of negative self-evaluation, and not the other domains of depressive symptoms or symptoms overall. These results underscore the complexity of stress sensitization and suggest that the relationship between negative events and specific depressive symptom domains may differ for girls and boys in ways that could inform prevention among at-risk youth. ELA moderated the between-person association between negative events and depressive symptoms such that in the context of higher average negative events, adolescents with low ELA reported higher depressive symptoms. Participants in this sample with high ELA did not show this association between negative events and all depressive symptom domains. Rather, ELA moderated the association between negative events and three depressive symptom domains (dysphoric mood, anhedonia, somatic complaints), but not negative self-evaluation, suggesting very little symptom specificity to the effect. Overall, and inconsistent with prior studies in adults^17^, there was little evidence in the present data to support the idea that ELA sensitizes adolescents to depression when faced with ongoing stressors. In other words, ELA did not moderate the association between negative events and increasing symptoms across the follow-up period. Instead, the present data converge with epidemiological studies showing that there is a diminishing effect of each additional stressful event on psychopathology^18,49,50^. To this point, it is important to note that ELA was associated with higher total depressive symptoms, particularly somatic complaints and negative self-evaluation. Thus, it is not the case that ELA-exposed adolescents in this sample were particularly resilient to depression, just that their depressive symptoms did not increase as a function of negative events. This raises the possibility that the patterns observed in the present data reflect differences in developmental trajectories, such that stress sensitization occurred earlier in the life span among high ELA-exposed youth than their low ELA-exposed peers. It may be helpful to remember that there are important, but under-appreciated, differences between stress sensitization, which was the hypothesis tested in the present study, and stress sensitivity^51^.

Briefly, stress sensitivity pertains to trait-like differences in the biobehavioral effects the environment has on an individual, and stress sensitization pertains to a process through which stressful experiences change an individual. The dynamic process of stress sensitization among ELA-exposed youth may need to be assessed in childhood rather than adolescence. Alternatively, individuals with and without ELA exposure may arrive at depressive symptoms and disorder via different trajectories. Indeed, ELA-exposed adolescents showed a general pattern of increasing depressive symptoms across the follow-up period independent of ongoing negative events. By contrast, adolescents in the sample with low ELA-exposure only reported increasing depressive symptoms in the context of negative events. Thus,  the pathway to depressive symptoms and disorder among youth without ELA-exposure fits the stress sensitivity model while the pattern among ELA-exposed youth does not. This may have broader implications for developmental psychopathology research aimed at better understanding distinct trajectories among vulnerable populations. To this point, an important next step is to examine when and how strongly negative events exert their effects. The negative event checklist captured occurrences but not timing (e.g., time-since-event, pubertal stage at exposure) or intensity, precluding more nuanced tests of developmental sensitivity. However, the present findings provide preliminary evidence that studies with a similar prospective, longitudinal design would benefit from incorporating time-stamped events, severity/chronicity ratings, and repeated pubertal assessments to evaluate whether exposures closer to assessment, during specific developmental windows, or of higher intensity are differentially linked to depressive symptom profiles.

### Limitations

The current findings are contextualized by several limitations. First, the sample size of 97 adolescents may have been insufficient to detect small effects, particularly whether minor within-person fluctuations in negative events across the year predicted variability or change in depressive symptoms. This possibility is even more likely given that there was limited within-person variability in negative events in this particular sample, which may have been influenced by the COVID-19 pandemic. Approximately 24% of assessments occurred during the first year of the COVID-19 pandemic (between March 11, 2020–2021). Although we found no evidence that there was a greater prevalence of total negative events at assessments which occurred during the pandemic (effect of COVID-19 on intercept *b* = 0.21 (*SE* = 0.37), *95%CI*[-0.51, 0.93] and slope *b* = -0.03 (*SE* = 0.04), *95%CI*[-0.51, 0.93]), it is possible that COVID-19 increased the prevalence of certain types of events (e.g., personal or family illness, financial strain) while decreasing the prevalence of other types of events (e.g., common academic and social stressors). For example, adolescents are particularly sensitive to social stressors among peers^52,53^, which may have occurred less frequently during the COVID-19 pandemic due to stay-at-home orders. There may also be limitations to the generalizability of findings garnered from this sample due to the recruitment approach. While the recruitment procedures in this study were designed to oversample for adolescents with ELA exposure using best practices for enrolling vulnerable youth who are historically under-represented in biomedical research^54^, the sample was not truly random or representative. In particular, the mass mailing invitations sought to reach a wide range of potential participants, but only parents who contacted the team after receiving this invitation were eventually enrolled. This may have created a bias in the sample that must be accounted for when generalizing these results to the population. Finally, sex was assessed using a single, self-report item that did not allow analyses to distinguish between sex and gender identity. This measurement limitation should be considered when interpreting the results pertaining to moderation by sex showing that negative events associated with greater negative self-evaluation scores among females, but not males. Nonetheless, this study had several strengths, including its longitudinal design with multiple assessments over 12 months. This design, coupled with the use of multilevel models, are the best approximation of causation possible in studies of ecological stress and depression. Further, the study advances dimensional understanding of depression and its symptoms, particularly among individuals known to be at greater risk for depression-onset in adolescence (females and the ELA-exposed).

In conclusion, the present data highlight important nuances to adolescent depression that can guide translation of prospective, longitudinal studies such as this one into intervention development and practice. In particular, dysphoric mood and somatic symptoms increased across adolescence, but negative self-evaluation was the symptom domain most sensitive to ongoing stressful life events, particularly among females. Prevention and intervention programs among at risk youth that target these symptom domains may be particularly efficacious in mitigating risk for depression-onset during adolescence. Further, depressive symptoms were more closely linked to ELA than ongoing stress, confirming the need to increase targeted interventions among ELA-exposed youth independent of their current stress exposures.

Depression in adolescence is associated with nearly a 3-fold increased risk of depression recurrence in adulthood^1^, as well as elevated risk of developing other psychopathology such as anxiety disorders, bipolar disorders, and suicidal ideation^1^. Adolescent depression also confers vulnerability for poor physical health, including a 70% increased risk of becoming obese compared to those who are not depressed^55^, and increased risk of cardiovascular disease^56^. Beyond adverse mental and physical health outcomes, adolescent depression impedes social functioning well into adulthood, including loneliness, unemployment, lower romantic relationship quality, and educational attainment^57–59^. Thus, mitigating risk for depression during adolescence is critical to improving public health in the broadest sense. These results suggest that focusing prevention and early intervention efforts on females demonstrating symptoms of negative self-evaluation and adolescents with a history of ELA may be essential to achieving this goal.

## Data Availability

The data that support the findings of this study are not publicly available due to the sensitive nature of the information that could compromise the privacy of research participants and their families. However, data are available from the corresponding author upon reasonable request and with the permission of the institutional ethics committee.
